# LionVu 2.0 Usability Assessment for Pennsylvania, United States

**DOI:** 10.3390/ijgi9110619

**Published:** 2020-10-23

**Authors:** Nathaniel R. Geyer, Fritz C. Kessler, Eugene J. Lengerich

**Affiliations:** 1 Department of Public Health Sciences, Penn State College of Medicine, Penn State University, Hershey, PA 17033, USA; 2 Department of Geography, College of Earth and Mineral Sciences, Penn State University, PA 16801, USA; 3 Penn State Cancer Institute, Hershey, PA 17033, USA

**Keywords:** usability assessment, web GIS, cancer, service area, geospatial health

## Abstract

The Penn State Cancer Initiative implemented LionVu 1.0 (Penn State University, United States) in 2017 as a web-based mapping tool to educate and inform public health professionals about the cancer burden in Pennsylvania and 28 counties in central Pennsylvania, locally known as the catchment area. The purpose of its improvement, LionVu 2.0, was to assist investigators answer person–place–time questions related to cancer and its risk factors by examining several data variables simultaneously. The primary objective of this study was to conduct a usability assessment of a prototype of LionVu 2.0 which included area- and point-based data. The assessment was conducted through an online survey; 10 individuals, most of whom had a masters or doctorate degree, completed the survey. Although most participants had a favorable view of LionVu 2.0, many had little to no experience with web mapping. Therefore, it was not surprising to learn that participants wanted short 10–15-minute training videos to be available with future releases, and a simplified user-interface that removes advanced functionality. One unexpected finding was the suggestion of using LionVu 2.0 for teaching and grant proposals. The usability study of the prototype of LionVu 2.0 provided important feedback for its future development.

## 1. Introduction

**Figure 1.**
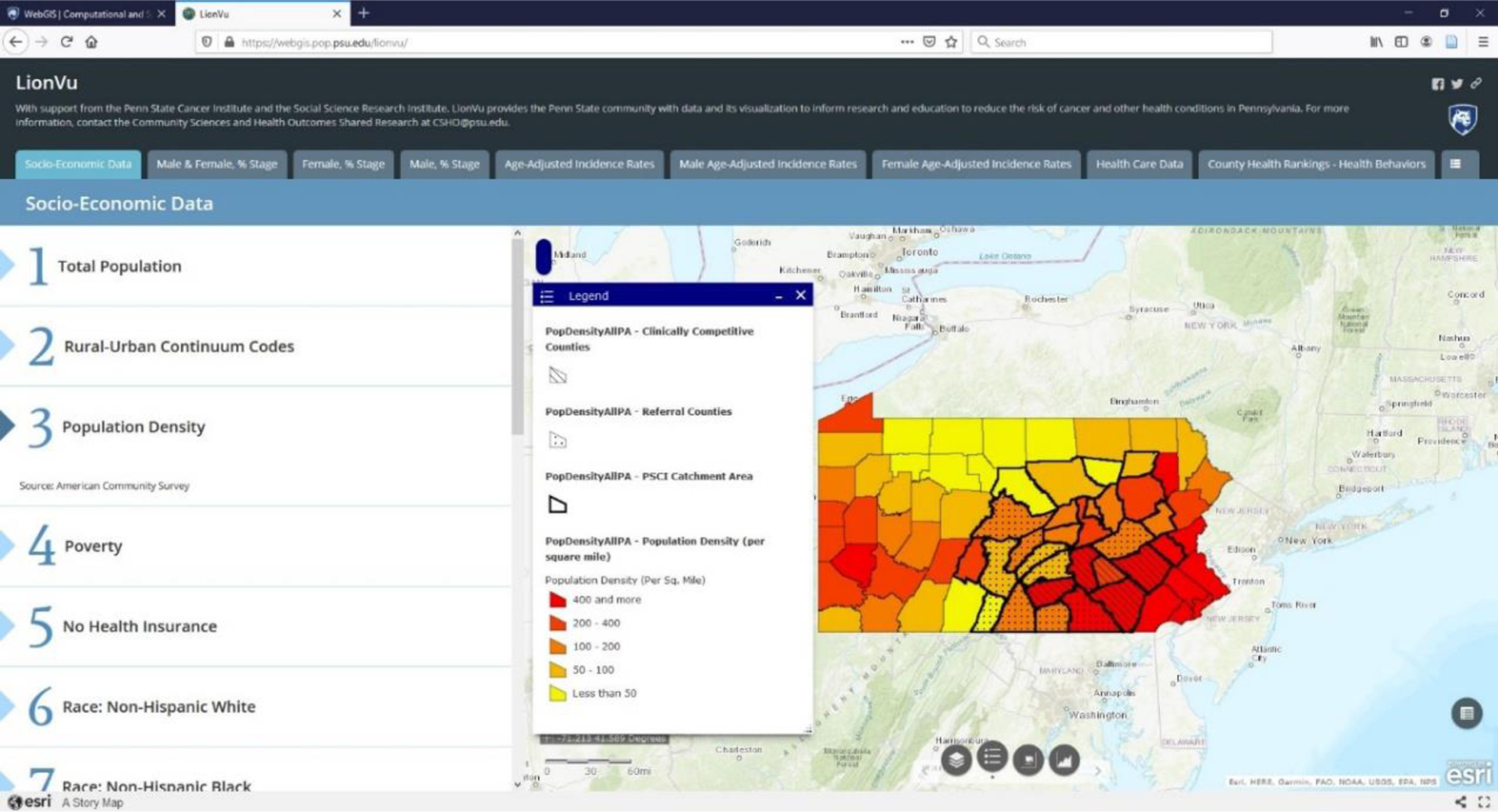
Original Design of LionVu 1.0 for population density (per square miles) within Pennsylvania. The 28 county Catchment Area is outlines in a thick black line.

In 2015, the Penn State Cancer Initiative (PSCI) implemented LionVu 1.0, a web-based mapping tool (Figure 1). LionVu 1.0 was designed to educate and inform the public health professionals about the risk of cancer within 28 counties located in central Pennsylvania, locally known as the catchment area. More specifically, LionVu 1.0 was designed to help answer research questions regarding the person–place–time nature of epidemiology related to cancer. The original targeted audience for LionVu 1.0 was PSCI health practitioners such as program managers, epidemiologists, and clinicians, but not the general public. The data sources used in LionVu 1.0 came from the United States Census Bureau, Pennsylvania Department of Health, and the County Health Rankings.

LionVu 1.0 has received criticism from users within PSCI, including not addressing the needs, i.e., of the PSCI catchment area and the lack of help documentation. These issues rendered LionVu 1.0 as unsatisfactory, with the need for revisions. Other criticisms focused on the user interface design, which permitted only one map to be displayed at a time. This map design was criticized for not using ColorBrewer 2.0 (Penn State University, United States) color scheme standards, which were developed to aid in visualization. Another criticism was the lack of comprehensive help documentation and tutorials for how to navigate through LionVu 1.0.

**Figure 2.**
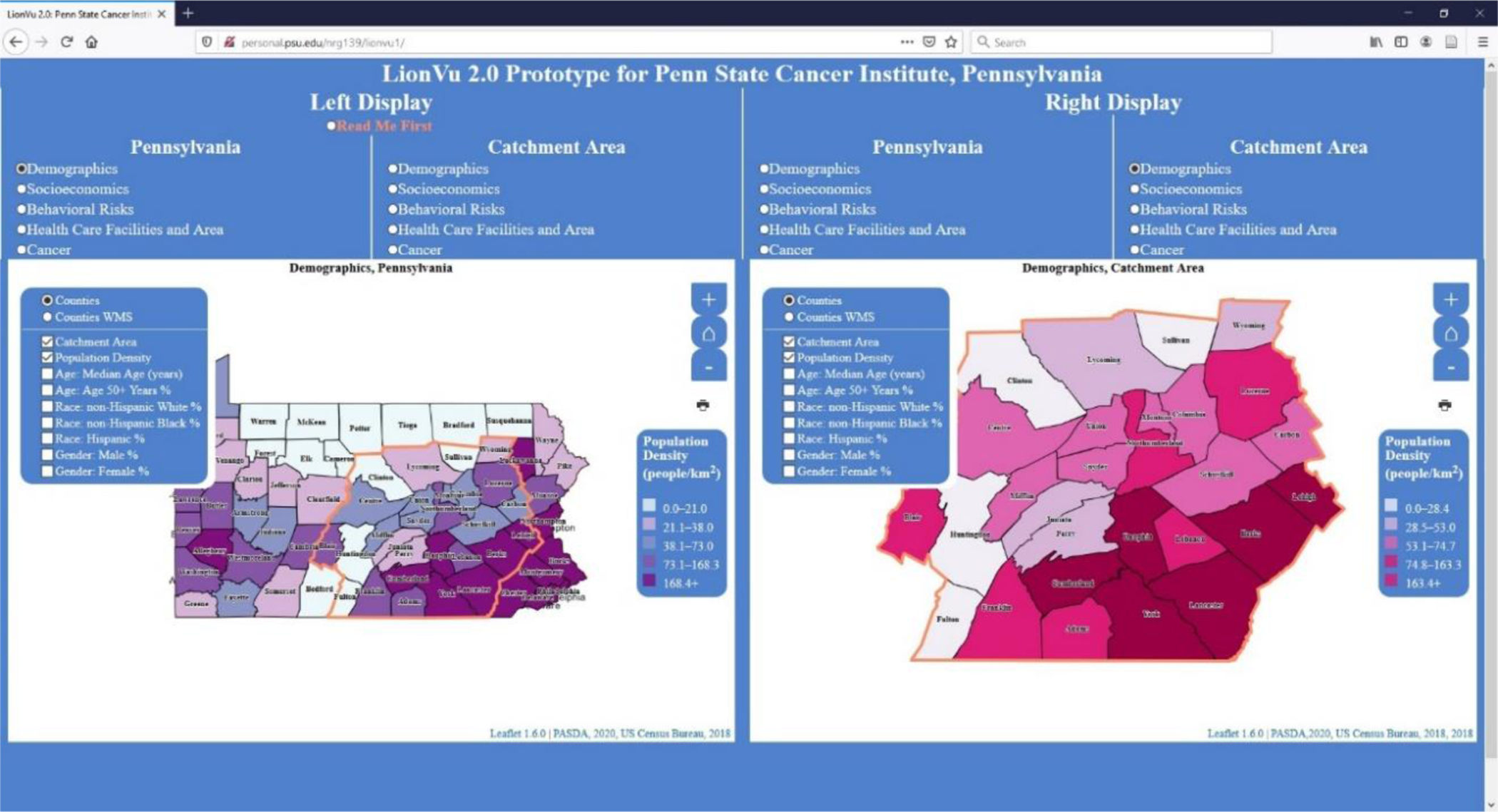
LionVu 2.0 used in usability assessment. Left Display: population density (people/km^2^) within Pennsylvania. Right display: population density (people/km^2^) within catchment area. Layer list was not collapsed in order to clarify what fields were available for selection.

In response to criticisms of LionVu’s 1.0 user interface, the development of LionVu 2.0 began in 2020. The criticisms of LionVu 1.0 were used as a framework guiding the redesign of LionVu 2.0, which included the following issues: mapping Pennsylvania and the catchment area side-by-side, updating the datasets, and including additional functionality, such as the ability to print. Figure 2 shows a redesigned LionVu 2.0 interface where Pennsylvania and the catchment area are mapped side-by-side. After exploring various data sources for Pennsylvania, revised data from the United States Census Bureau, Pennsylvania Department of Health, County Health Rankings, and Medicare were used. Advanced functionality, such as the ability to print and help documentation (i.e., read me first section) and color schemes based on ColorBrewer 2.0 were added. Based on the recommendations of Brewer and Pickle, the quantile classification was used as the default classification, since it had the most accurate epidemiological mapping applications [1]. Once initial criticisms were addressed in a prototype, a usability survey was conducted to guide the further development of LionVu 2.0, which is the focus of this paper.

### 1.1. Literature Review

The focus of this literature review was to examine research on usability assessment methodologies, focusing on the health and geospatial disciplines, to help develop the LionVu 2.0 usability assessment. We searched three databases: PubMed, Web of Science, and Cochrane for usability assessment methodology articles, using keywords: usability study, user feedback, (GIS) systems design, prototyping, web mapping, spatial analysis software, cancer, health, ecology, participants as investigators, usability metrics, and policy makers. Google Scholar’s cited-by function was also employed to find additional sources published between 2015 and 2020. Seminal work that was published prior to 2014 was also reviewed.

Usability studies in the health field included methodologies such as concordance or consensus building [2,3], discordance or tension [4], qualitative weight and sum method [5], mixed methods [6], focus groups [7], or surveys [8]. One of the more relevant investigations was a two-part study that analyzed existing Missouri printed maps (InstantAtlas) in a web mapping tool, which were developed by the Missouri Department of Health [9,10]. This usability assessment was conducted during two time periods with part one being completed by a subset of college students first, followed by a second usability assessment including a subset of health professionals. The two-part usability studies of InstantAtlas were designed to evaluate user friendliness and user satisfaction, and test whether the usability improved by modifying InstantAtlas web maps on an ongoing process comparable to our development of LionVu 2.0. Based on the results of part one, the InstantAtlas web map was revised and the new version went through a second usability assessment in part two. A select group of policymakers and clinicians examined InstantAtlas in part two to determine if the modified maps improved the usability according to the suggestions of part one. Part two found that the InstantAtlas web maps were effectively utilized by participants with limited mapping experience. Some additional suggestions were to simplify the printed maps and improve the help documentation on how to interpret the information in the web maps.

Geospatial usability assessments are critical for determining the quality of a user’s interaction with internet-based mapping applications and measuring how accessible web maps are to participants. One study used eye-tracking to measure how well a mapping prototype, mimics real-life applications, stressing the importance of a properly designed user interface when completing tasks online [11]. Web maps that went through a usability assessment also need to have responsive web design that works in all platforms: desktop, mobile, and laptops, while balancing customization of functionalities [12]. In addition to a well-defined user interface, it is also critical for using a survey instrument (i.e., system usability scale (SUS) and participatory GIS usability scale (PGUS)) to insure that the scale can differentiate between usable and unusable systems [13,14]. The SUS uses 10 Likert scale-based questions that are easy to administer with small sample sizes and can effectively differentiate between usable and unusable systems [13]. The PGUS uses 25 Likert scale-based questions in five domains (i.e., user interface, spatial interface, learnability, effectiveness, and communication) and seven demographic and participants questions [14]. The PGUS was first used in a marine spatial planning group and was more applicable to geospatial usability assessments than SUS and can better facilitate rapid evaluation for web mapping projects.

Key components of the usability assessment were an evaluation of completion time, efficiency, and effectiveness. Measures to evaluate the effectiveness include baseline performance usability metrics such as satisfaction, efficiency, and effectiveness [15]. Effectiveness is the ratio of task completion to number of errors. Efficiency is the ratio of task completion to task time in minutes [16]. Although these studies focused on efficiency and effectiveness there was little to no assessment on learnability, which was measured as part of the PGUS survey questions.

Based on the results of this literature review, several themes emerged that focused on: (a) having a well-designed user interface; (b) displaying visually appealing maps; (c) using a usability assessment scale (i.e., SUS and PGUS); and (d) using a survey with both open-ended and close-ended questions. When developing the usability assessment, we started with the PGUS survey tool. The PGUS contains seven sections and roughly 30 questions on demographic characteristics, participant’s characteristics, and five Likert-scale sections (i.e., user interface, interaction, learnability, effectiveness, and communication). For our assessment, we adapted a total of 20 PGUS Likert-scale questions from Ballatore et al. [14]. Specifically, we adapted five PGUS questions on demographic characteristics (i.e., gender, race, ethnicity, age, and educational attainment) and four on employment characteristics. One question from each of the five Likert-scale sections was selected to evaluate the user interface, accessibility of information, how to perform tasks, reliability, and whether the maps are easy to understand was included as individual tasks. The LionVu 2.0 usability assessment built on these themes assessed how well the user interface displays visually appealing maps, to an audience with limited mapping experience.

### 1.2. Objectives

The primary objective of this study was to conduct a usability assessment of a prototype of LionVu 2.0 using domain expertise related to cancer data. To complete the usability assessment, we developed 50 survey assessment questions, based on the literature review section, including both open- and close-ended questions. The usability assessment was distributed via REDCap (Research Electronic Data Capture) survey system. The qualitative survey data was analyzed in two ways: content analysis and item analysis for evaluating patterns in survey data (i.e., task completion, effectiveness, efficiency, and time). A discussion of key findings from the usability assessment will be addressed, including limitations, recommendations, and conclusions.

## 2. Materials and Methods

After reviewing the various methodological approaches, we decided that a survey was the preferred method to reach participants from various organizations and geographic areas. We targeted professionals located in the 28-county catchment area. Since the professionals were spread out in organizations throughout the 28 counties, the survey had to be conducted asynchronously with participants being given the opportunity to do the assessment in multiple settings.

### 2.1. Survey Question Development

The online survey of LionVu 2.0 contained 10 sections: (1.) Demographics Characteristics; (2.) Employment Characteristics; (3.) United States Cancer Statistics (USCS) Web Map; (4.) Task 1: Demographics; (5.) Task 2: Cancer; (6.) Task 3: Health Care Facility and Area; (7.) Task 4: Behavioral Risk and Socioeconomic; (8.) Likert Scale; (9.) Purpose, Data, Help Documentation, and Functionality Feedback Questions; and (10.) General Feedback. Each had five questions, for a total of 50 questions. In addition to tallying responses to the 50 questions, time for completion was recorded for each participant on 10 of the questions. The survey was expected to be completed in 30–45 min. We discuss the survey question development and REDCap and ethical considerations in separate sections.

This survey contains 10 sections with 5 questions each totaling 50 questions (Table A1 in Appendix A). In the demographics section we asked the participants about gender, race, ethnicity, and highest educational attainment. In the employment characteristics section, we asked the participants about employment and a self-reported web-mapping proficiency question based on the National Institutes of Health’s Competencies Proficiency Scale [17]. In the USCS web map section, we asked the participants five task-specific questions about a Centers for Disease Control and Prevention web map [18]. The first task-related question prompted the participant about the 5-year time period for new cancer diagnoses in Pennsylvania based on the USCS web mapping environment. The four other questions asked the participants about the strengths, limitations, expectations, and capabilities of the USCS map. The purpose of this section was to orient the user about other web maps that were outside of our control. The four other task-related sections involve assessing the participant on specific tasks using the LionVu 2.0. For example, in task 1, we asked the participants to determine population density and Hispanic population in Luzerne County. In this task, the population density required the user to select demographic layers from LionVu 2.0’s user interface and to respond to multiple-choice questions. There were three additional open-ended questions on determining how the user was able to find the answers and applying the results to the entire state of Pennsylvania and the catchment area. Other task-related sections asked users to interpret cancer data, to identify health care providers using point data, and to evaluate the side-by-side functionality. The intent of these task-related questions was to ensure that LionVu 2.0 was comprehensively reviewed. The Likert Scale section asked the participant questions based on the five target areas of the PGUS (i.e., user interface, interaction, learnability, reliability, and communication). The last two sections asked the participant open-ended questions about the general purpose, help documentation, functionality, and general feedback questions about areas of improvement about the LionVu 2.0.

### 2.2. REDCap and Ethical Considerations

We collected survey data for the usability assessment and managed using REDCap, a secure web application designed to support data capture for research studies. The advantage of REDCap over other survey systems is that it allows for surveys to be ethically compliant and for participants to finish where they started. Doing so provides a user-friendly web-based platform to capture and validate data for quality assurance purposes. The data used for this survey are hosted at the Penn State Health and College of Medicine data center. The data are protected under the Health Insurance Portability and Accountability Act of 1996 (HIPAA), which is protected from disclosure without consent. REDCap gives the investigator the opportunity to download a statistical software and do more complex analyses [19]. REDCap also allow us to preload a list of emails for invitations to send to participants asking to complete a survey. Using the REDCap system for this usability assessment required a review by the Penn State College of Medicine’s institutional review board (IRB) and was determined to be non-human subjects research (IRB # STUDY00015009).

### 2.3. Sample Population and Procedures

We initially sought a response rate of 33% from a sample frame of 25 PSCI members and 20 people from the Pennsylvania Department of Health. The initial invitation was sent on 13 May 2020. On 18 May, we sent an invitation to the entire membership of the PSCI cancer control program, as requested by the director of the PSCI. On 15 June, we closed the survey with 23 responses, 10 of which completed the entire survey.

### 2.4. Analyses Performed

We performed a content analysis of participant responses to all open-ended questions that were reported on the usability assessment. We also performed an item analysis of participant responses to the closed-ended questions identifying skewness of the demographic characteristics, employee characteristics, and accuracy measurements for the task related questions. The time intervals of completion time were estimated using a web calculator [20]. In addition, we analyzed and reported the measures of effectiveness and efficiency using the definitions adapted by Gómez Solórzano et al. [16]. Given the small sample size for this survey, there was not enough statistical power to perform complex analyses, but it did help inform the future directions for developing LionVu 2.0.

## 3. Results

We sent invitations to the LionVu 2.0 usability survey to 123 individuals; 23 started and 10 completed the survey. The response rate was 19% and completion rate was 43%. The completion time (geometric mean: 51.41 min; median: 50.5 min; 95% Confidence Interval: 37, 71.5) was over the 30–45 min time estimate, which was adjusted for people who stopped and came back to the survey at a later time period. The results discussion is organized based on the 10 sections of the survey, with an additional summary that reported the effectiveness and efficiency of LionVu 2.0.

### 3.1. Demographic Characteristics

We asked five demographic questions about age, race, ethnicity, gender, and highest educational attainment. The median age of the subset was 56 years. The self-reported race, which was defined using the United States Census Bureau’s categories, was 71% White, 19% Asian, and 10% Black. The self-reported ethnicity, as defined by the United States Census Bureau was 15% Hispanic. The self-reported gender was 55% female. The highest educational attainment earned was 65% Doctorate (e.g., PhD, EdD, DrPH), 15% Professional (e.g., MD, DDS, DVM), 15% Masters (e.g., MA, MS, MEd, MPH), and 5% Bachelors (e.g., BA, BS) degrees.

### 3.2. Employment Characteristics

We asked the users to identify their work location, type, organization, title, and proficiency of web mapping. The employment location was 65% Hershey, 20% state college, and 15% Harrisburg; work type was 70% faculty, 15% manager, 10% clinician, 5% staff; while the organization type was 45% Penn State College of Medicine, 20% Penn State Health, 20% Penn State University Park, 10% Pennsylvania Department of Health, and 5% Penn State Harrisburg. Common work titles included: 42% assistant professor, 31% professor, 7% administer, 5% program director, 5% research project 5% manager, and 5% physician. The web map proficiency was found to be 45% fundamental awareness (e.g., beginner), 25% limited experience, 20% intermediate, 10% advanced, and 0% expert. In addition, we had 20 people who completed the questions in this section, who were grouped based on job title to either academics or professionals, which were evenly split at 10 for this section. In the following sections there was non-completion in roughly half of the participants leading to a sample of six academic and four non-academic professionals by the time the Likert scale questions were asked.

### 3.3. United States Cancer Statistics (USCS) Web Map

These questions asked participants to visit the USCS web map to determine the 5-year age-adjusted rate of new cancers in Pennsylvania [18]. The response accuracy to the 5-year rate of new cancers, was low (13%). This low accuracy was largely due to the poorly designed web map interface which displayed the 1 year rate by default, while the survey instructions required the participant to click an obscure radio button to change the map to the 5 year timeframe. Other limitations reported by the participants were poor data availability (i.e., data were out of date), hard to navigate, and the interface was not intuitive. On the other hand, reported strengths of the web map from the participants included: the ability to manipulate data and easy interpretation the web map. To most of the participants the USCS’s web map met their expectations, but some individuals noted the limited capability to map data at the state level.

### 3.4. Task 1: Population Densities and Hispanic Populations within Luzerne County

**Figure 3.**
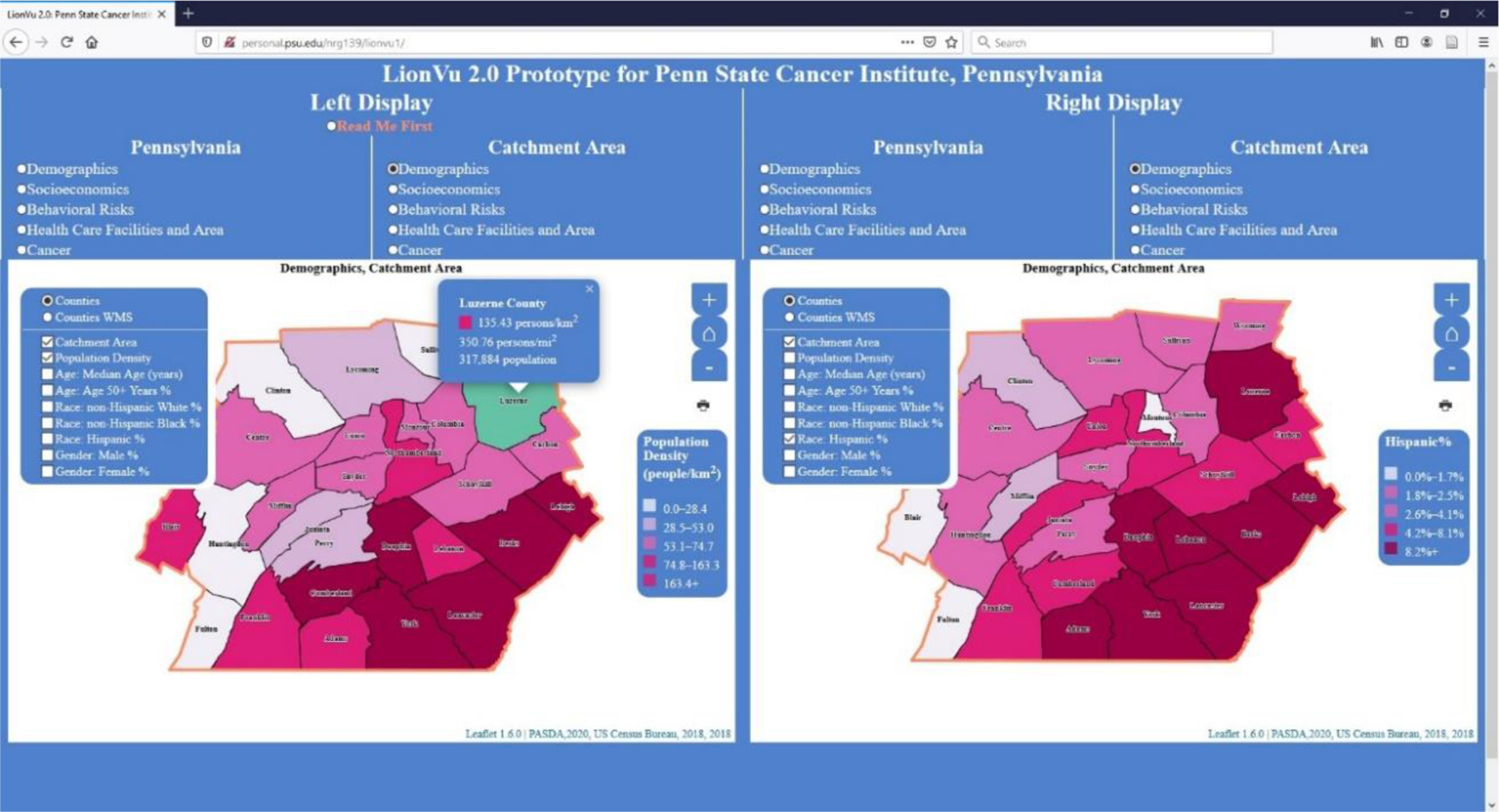
Screenshot of task 1. Left display: Population density (people/km^2^) within catchment area. Right Display: Percent Hispanics residing within catchment area. Layer list was not collapsed in order to clarify what fields were available for selection.

In task 1, we asked the participants to use choropleth maps to determine the population density and color value for Hispanics in Luzerne County (Figure 3). The response showed that 92% answered the population density question correctly, but only 58% provided the correct color value. This low accuracy on identifying the color value was due to a contrasting blue background on the legend, white on the map, and yellow on the survey. In the REDCap survey, participants were given a multiple-choice answer with various color values and asked to click the answer that best fit the situation. The wording of the task asking about the color value could have been confusing to the participants. The intent was to have the participant match the color value on the map to the data class on the legend. One positive comment was that the visualization of the choropleth maps appeared to show an increased population density in Philadelphia and Pittsburgh. One negative comment was the inability to find help documentation (i.e., read me first section).

### 3.5. Task 2: Assessing Cancer Patterns

**Figure 4.**
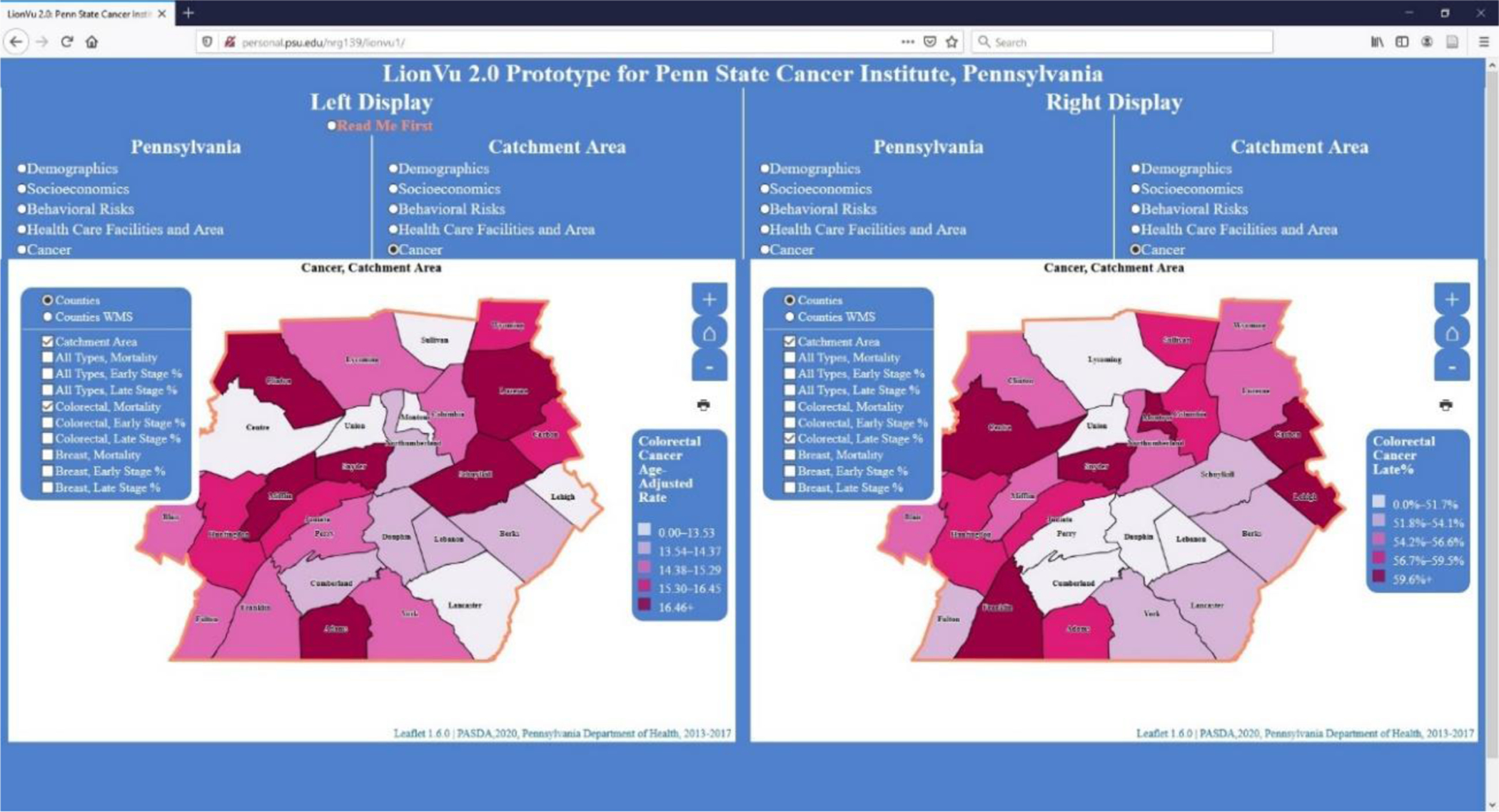
Screenshot of task 2. Left display: Colorectal cancer mortality age—adjusted rate within catchment area. Right display: Late stage incidence percentage of colorectal cancer within catchment area. Layer list was not collapsed in order to clarify what fields were available for selection.

In task 2, we asked participants to use choropleth maps to determine the mortality and percentage of colorectal cancer in the Centre County that were diagnosed at a late stage (Figure 4). The participants answered the two cancer-related questions (92% mortality; 100% late stage percentage) correctly. This high rate of accuracy was likely because most participants were from the cancer field, which made them more critical towards the quality of LionVu 2.0. This task was praised for the maps and including a variety of cancer mortality and stage information for colorectal, breast, and all cancers. However, the web map was criticized for the lack of data on cancer survivorship and other types of cancer.

One map design issue had to do with the county labeling. The Leaflet JavaScript programming language prevented the labels from being added directly to the web map. In the LionVu 2.1, labels were fixed by using the QGIS 3.14 centroid function and then created a tooltip at the centroid to show the county name. Another issue was the lack of user control over classification methods (i.e., quantile, natural breaks, etc.) between the Pennsylvania and catchment area maps. One participant asked for the option to change the color scheme and include the option to export images.

### 3.6. Task 3: Health Care Facility and Area

**Figure 5.**
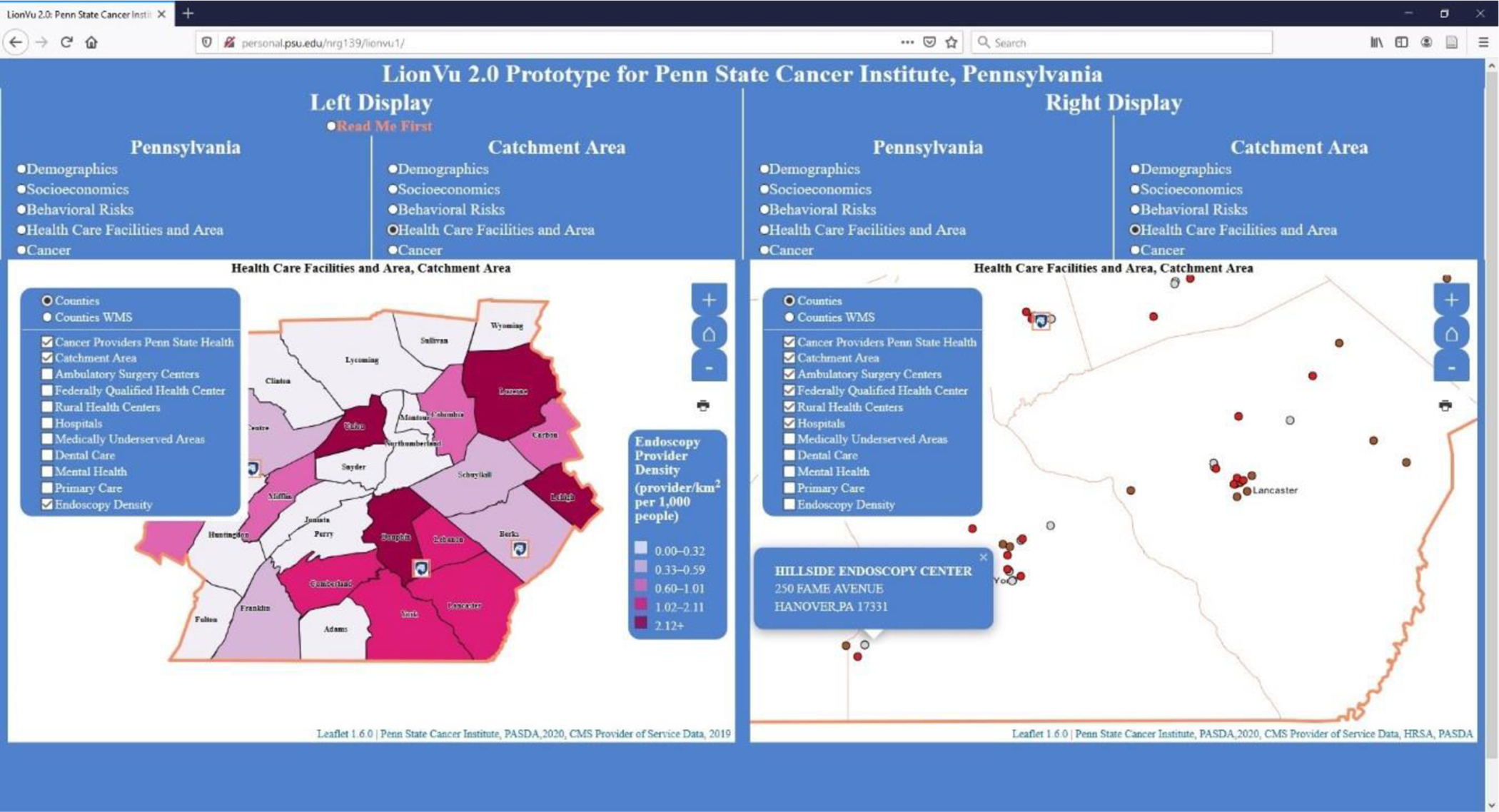
Screen shot of task 3. Left display: Endoscopy density (providers/km^2^ per 1000 population) within catchment area. Right display: Health care providers (dots: Gray-Ambulatory Service Centers; Brown-Federally Qualified Health Centers; Red-Hospitals) within catchment area. Layer list was not collapsed in order to clarify what fields were available for selection.

In task 3, we asked participants to review choropleth maps to determine the density of endoscopy providers in Lancaster County. Participants were also asked to examine a dot map and indicate the number of providers that were not found in Lancaster County. Of the responses, 90% answered the density of endoscopy question correctly, but only 30% correctly identified Hillside Endoscopy Center which is found in York County (Figure 5). The reason for the 90% accuracy was that endoscopy density is not a widely used measure of density, but since it was a choropleth map it was easy to figure out the density by hovering the mouse over Lancaster County. Whereas the low accuracy of identifying Hillside Endoscopy Center was because the participant had to hover the mouse over each point and view the pop-up, which was confusing and hard to identify. In order to rectify this problem, it was requested to include tabular data, instead of hovering over each point. Part of the technological hurdle here is that the Leaflet JavaScript does not include tabular outputs, so additional plug-ins are needed, which will increase the time needed to process and load the map by the browser. We included tabular data and the option to print the map into the tool. However, the download data were found in the read me first section (e.g., the location was not evident to the participants), and the print button on the map was difficult to locate.

### 3.7. Task 4: Behavioral Risk and Socioeconomics

**Figure 6.**
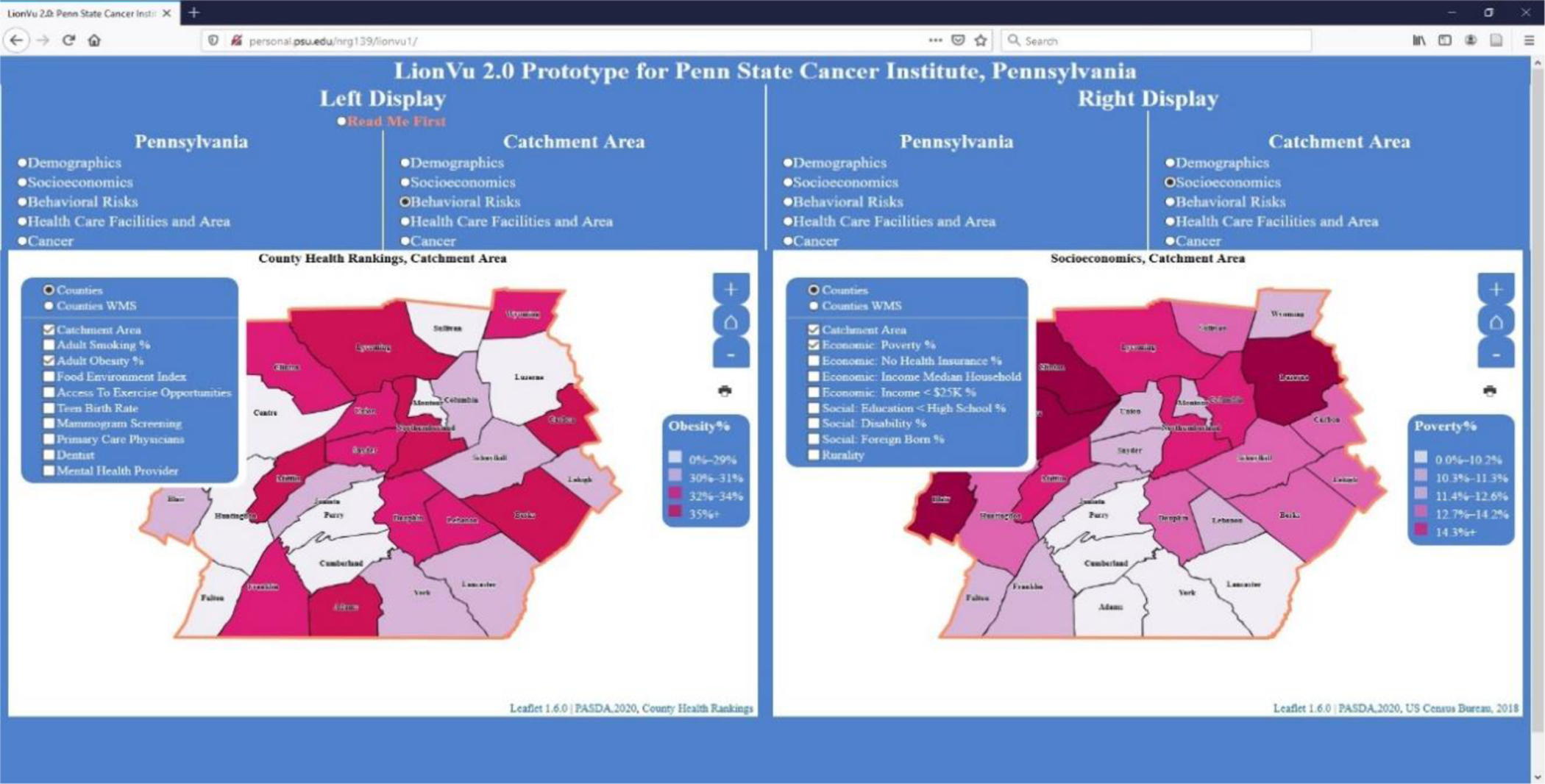
Screen shot of task 4. Left display: Obesity percentage within catchment area. Right display: Poverty percentage within catchment area. Layer list was not collapsed in order to clarify what fields were available for selection.

**Figure 7.**
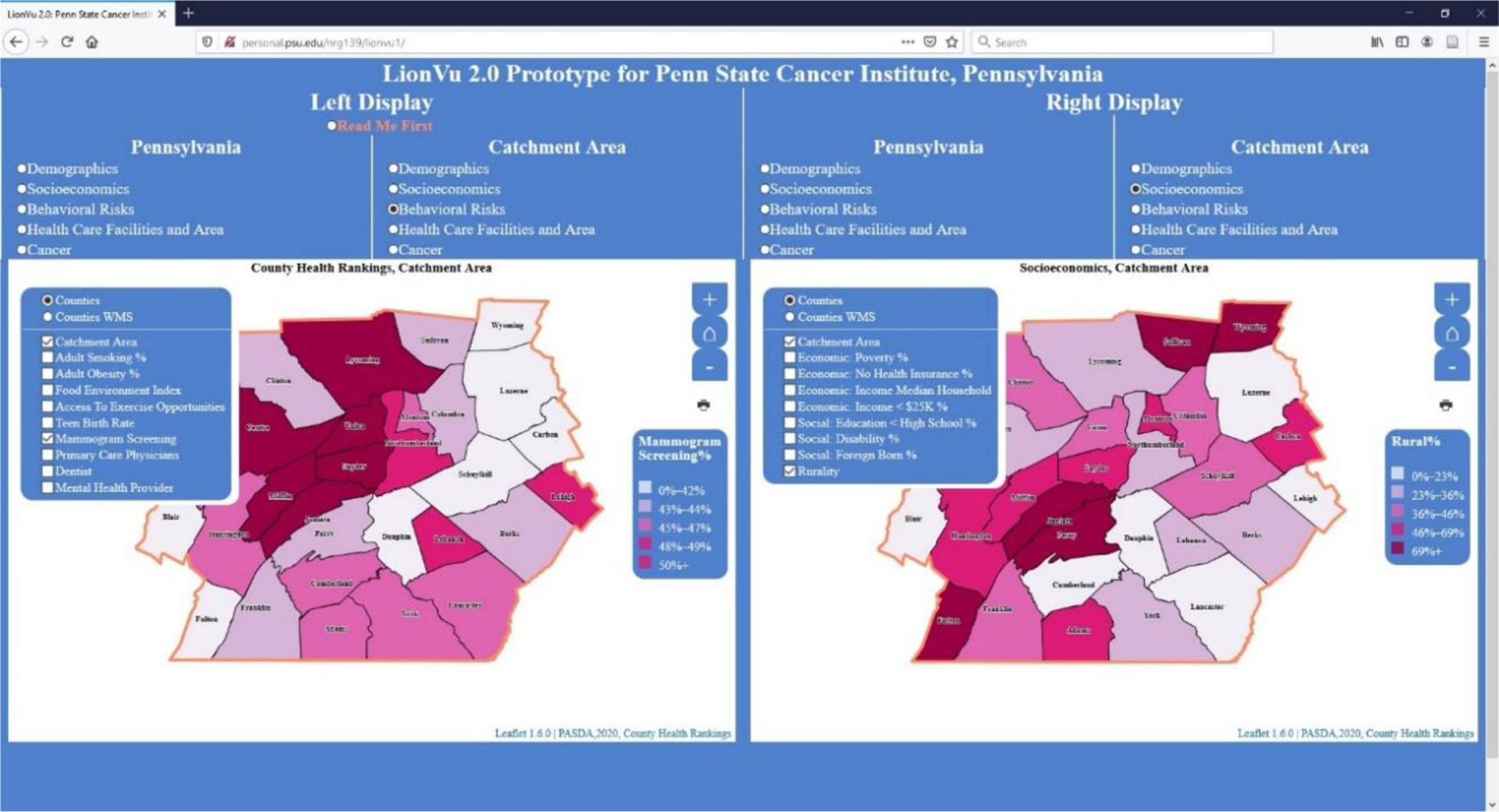
Screen shot of task 4. Left display: Mammogram percentage within catchment area. Right display: Rural percentage within catchment area. Layer list was not collapsed in order to clarify what fields were available for selection.

In task 4, we asked the participants to use the side-by-side displays of choropleth maps to measure the functionality by comparing two maps of selected characteristics, within Centre and Luzerne Counties. A hint was provided to the participants to use both displays with the left window displaying the behavior risk characteristics for the catchment area, while the right window displaying the socioeconomic characteristics. The first question asked participants about adult obesity % and poverty percentage in Luzerne County, resulting in 90% accuracy (Figure 6). The second question asked about mammogram screening and rurality percentages in Centre County, resulting in 80% accuracy (Figure 7). One reason for the 80–90% accuracy had to do with a participant using the legend instead of the popup to answer the mammogram and rurality questions. Another concern was the widespread distribution (0–23%) with the quantile data classification that resulted in Philadelphia having 0% rurality but being grouped in with the 0–23% group. There is an additional concern about the description of what selection was being measured, such as what do percentage of screening and rurality mean as displayed in the web map.

### 3.8. Likert Scale Questions

We used Likert scale questions to measure the participants on five target areas: user interface, interaction, learnability, reliability, and communication, with one being strongly disagree and four being strongly agree, as adapted from the PGUS scale [14]. The median score of the 10 participants was highest for both learnability (four) and reliability and lowest (three) for the user interface (Table 1). In addition, the professionals who have job titles other than professor or lecturers tended to rate LionVu 2.0 with a lower score than professors or lecturers did. One major concern with the user interface was the inclusion of the layer list, which was a checkbox, which allowed multiple choropleth layers to be selected, making the map difficult to interpret. Another concern was the over-sensitive ability to zoom in just by clicking the mouse (i.e., zoom by click). There were two general concerns with the interaction question. Here, participants asked for more help documentation to resolve the situation when there was a desire to return to the data source. Additionally, the quantile data classification method was used for each variable. This led to confusion with the participants who felt that switching to a Natural Breaks or another classification method would present the data in a different perspective. Two common concerns with learnability included not having any training videos recorded to aid in using LionVu 2.0 as part of the help documentation. Two focused concerns with reliability included a time delay between selecting a layer and labels as well as screen display glitches especially with the county labels.

### 3.9. Purpose, Data, Help Documentation, and Functionality Feedback Questions

In the next section, we asked participants to provide specific feedback on the purpose of LionVu 2.0, the timeliness and relevancy of the data, the help documentation usefulness, and the overall functionality. The participants considered the primary purpose of LionVu 2.0 to be: providing a quick view of local data, mapping preliminary data for grants, performing basic geographic analyses, visualizing existing data, and using the information for classroom discussion or other teaching purposes. The participants responded to two questions about the addition of more recent data on cancer survivorship and cancer types but did not ask to remove any data from LionVu 2.0. Participants provided comments stating that the help documentation read me first section was mostly ignored prior to reading this question. Possible ways to improve the presence of the help documentation would be to make it more persistent on the home page and the need to be comprehensive, and understandable to the participants. Suggested additional features in LionVu 2.0 include: exporting to an image file, including tables, putting the layer selection in a dropdown menu, and removing the ability to zoom-in the map by clicking the mouse.

### 3.10. General Feedback

In the final section, we asked participants about strengths, limitations, job applicability, and general comments about both the LionVu 2.0 and the survey instrument. One of the overall strengths was that participants felt that it was easy to visualize and learn about patterns in the data from the choropleth maps. Limitations were the user interface (i.e., radio buttons reduced the space of the displays making it difficult to see on smaller screens), lack of historical data to complete a multiple year assessment, difficulty in exporting an image from LionVu 2.0, and the collapsible layer list made it difficult to select and unselect layers. The lack of adequate training for LionVu 2.0 users meant there is a potential for future growth in this area. Responding to the question about whether LionVu 2.0 provides the tools to complete your job, most of the participants indicated that it did, but added the potential for teaching and classroom discussion. Other participants responded that it may not be applicable to their careers, but it was visually appealing and can be a good introduction to how to view other web maps. There also was a request for the addition of layers of information on county names, catchment area counties, and the Pennsylvania and catchment area counties found in Appalachia [21], which were added to the LionVu 2.1 version.

### 3.11. Usability Comparison

For the usability comparison, we defined an academic as people who have a job title of any type of professorship or lecturer, and non-professional being people who have any other job title outside of academia. The median time spent by participants to complete the usability assessment differed among academics and non-academic professionals. Of the 23 people who started the survey, 10 were from academia, 10 from professional organizations, and three with missing professional information. Table 2 shows the performance based on the median time in seconds, success rate, and sample size, for total, professional, and academic subsets. Based on the effectiveness non-academic professionals had a higher total effectiveness and efficiency than those who work in any academic positions (Tables 3 and 4). Based on the small sample size of the survey, the times were measured at the beginning of each section, so it is possible to analyze participants who did not complete the survey. The data were not normally distributed, so we report the median times instead of the average times.

## 4. Discussion

The original purpose of this usability assessment was to evaluate how successful the LionVu 2.0 was in serving the needs of PSCI health practitioners. In order to develop an effective web mapping tool for public health purposes, it is important to identify the critical content for facilitating participants’ decision making and to develop the optimal interface for ensuring ease of information access and usability [22]. Findings of the usability assessment indicated that most people had a favorable view of LionVu 2.0. While most participants had little to no experience with web mapping, it was not surprising to learn that participants wanted short 10–15 min training videos to be available with LionVu 2.1. One unexpected finding was the idea that LionVu 2.0 would be useful for teaching purposes and writing grant proposals. There was also a suggestion to simplify the interface by removing advanced functionality, which can be done by reducing the number of JavaScript functions thereby decreasing the time needed for the browser to process loading the maps [23]. Another suggestion was to allow the end user to change the data classification method (i.e., equal interval, natural breaks, etc.), color values, based on ColorBrewer 2.0, for the choropleth maps. An ongoing revision plan is to increase the participatory design through a mechanism that provides feedback such as a short survey that is linked to LionVu 2.1’s homepage.

### 4.1. Limitations

The limitations of this usability assessment impacted generalizability, validity, and reliability of the findings. First, the response rate of 19% was lower than the goal of 33%, most likely due to the start of the COVID-19 pandemic, which most likely reduced the response rate. Second, the low completion rate of 43% was due to the design of the survey with 50 questions that may have overwhelmed the participants who have busy schedules. This issue was partially mitigated by allowing the participant to come back later to complete the survey. In hindsight we realized that the survey took longer to complete than we anticipated, which diminished the response rate. A shorter survey could have provided a richer set of feedback. Third, while the survey was being administered, the USCS web maps were updated to newer data and removed the 2012–2016 maps from the website and replaced them with 2013–2017 maps, which had different data values. This issue was mitigated because most participants completed this portion of the survey prior to the USCS updates. A fourth issue was the large number of Department of Health employees with inactive email addresses, due to an outdated contact list which meant that many invited participants never received the formal invitation to participate. This issue was mitigated by the last-minute addition of 83 community members. Some of the challenges for effective evaluation of mapping tools include: (a) the lack of methods for testing usability, (b) analyzing complex workflows, and (c) assessing long-term usage [24]. Therefore, the results are only reflective of this assessment and cannot be validated or generalized to other evaluations. Despite these limitations, we believe that by implementing the recommendations in the next section will produce an improved LionVu 2.1.

### 4.2. Recommendations

**Figure 8.**
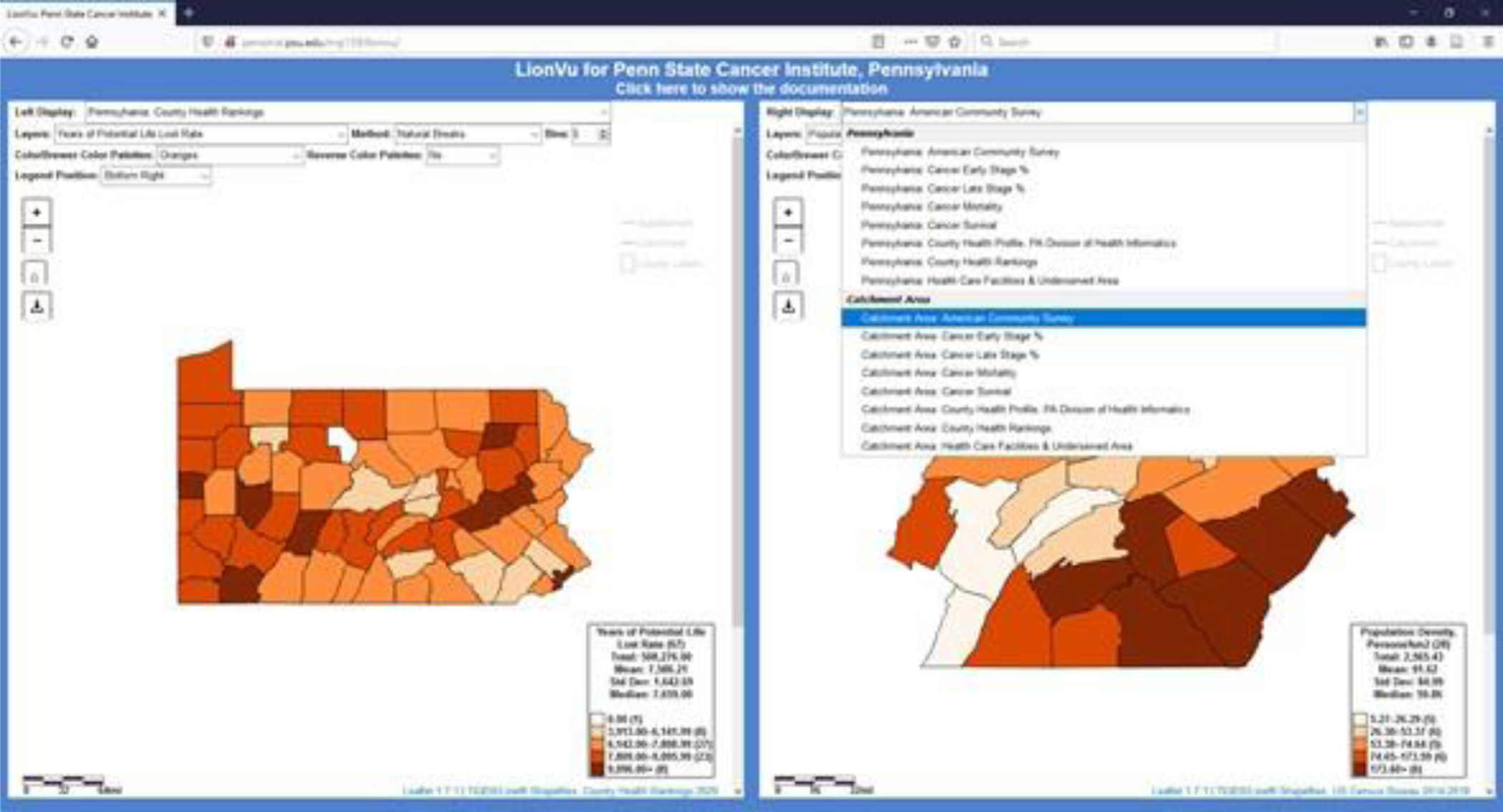
LionVu 2.1 tool using seven dropdown menus and a button to download maps, while preserving side-by-side displays. Left display: Years of potential life lost rate within Pennsylvania. Right display: Population density within catchment area.

There are seven recommendations to improve the LionVu 2.0 before and after the launch by December 2020. First, there is a need for developing action items for future programming efforts. Second, LionVu 2.1 will include functionality to allow the user to select data, method chose a color schema, modify the legend position, and set the number of data classes, using seven dropdown menus instead of checkboxes, and the addition of button to download the map as an image file (Figure 8). Third, the suggestion of using LionVu 2.1 in a classroom setting could be piloted into existing educational classes, which may expand the user base and offer additional recommendations for functionality not presently integrated into LionVu 2.0. Fourth, there is a need for moving to a server host that is HIPAA compliant rather than the Penn State access account storage space server where it presently sits. Fifth, development of short training videos needs to be completed prior to the anticipated December 2020 launch. Sixth, in LionVu 2.1, we created a separate data class that contained duplicates or rates that are equal to zero, as shown in the left display in Figure 8. Seventh, there is a need for users to see tabular outputs of the mapped data, using a jQuery plug-in DataTables, with buttons to export to comma separate values (.csv) file, adjust page lengths, and change column visibility.

As shown in Figure 8, in the case of years life lost rate, there was a request for five data classes and natural breaks classification, but the first class had a value of zero, which was assigned a white color and a duplicate class value, which was removed from the map. Whereas in the right display the population density value were neither non zeros nor duplicates so the default values of the five classes were mapped. This ensured that the end-user will not be seeing wide ranges or duplicate class values, which was a problem with LionVu 2.0. In addition, in LionVu 2.1, we implemented geostat.js and chroma.js for choropleth maps, which allows the end-user to switch layers of information, classification method, sequential ColorBrewer 2.0 color palettes, or add/remove/adjust the position of a legend. The default was set at five classes or bins, orange color palette, quantile method, and bottom-right legend. However, we did give the user the option to change layers of information; seven classification methods; 1–20 bins/classes; 18 ColorBrewer 2.0 sequential color palettes; option to reverse color palette (i.e., cancer survival uses inverted color palettes) and add, remove, or adjust the position of a legend. Additionally, in LionVu 2.1 we replaced the checkboxes with toggleable displays, using a leaflet JavaScript plugin [25], for Appalachian and catchment county overlays, and county names. Lastly, we also included tabular outputs (i.e., DataTables) below the maps that allow the user to view the data.

## 5. Conclusions

As noted by Dr. Gerry Rushton in 2003, the future of GIS in public health in the United States will be the development of specialized disease surveillance systems, clinical practice location will broaden to geographic areas, and public health personnel will need to be trained in the GIS environment [26]. In 2020 we are now using specialized surveillance systems, clinical sites are using web GIS, and people are being trained to use the data in web-based platforms. The challenge is that often the products are internally developed and are limited in substance or only perform specific analyses for a limited set of variables. As noted previously, the purpose of this usability assessment began with a very primitive web mapping tool. Unlike previous usability assessments, LionVu 2.0 is being constantly developed to allow for mapping comparisons for a variety of different variables and locations in each dataset for Pennsylvania. For example, the County Health Rankings, are published annually where only a selected number of fields are mapped, even though the datasets have more than 100 variables. In LionVu 2.1, all 100 variables are loaded to a file and presented on a map for both Pennsylvania and catchment area, side-by-side. In addition, other datasets used may be specific to a given geographic area, but the programming can be replicated to a larger area and datasets, potentially increasing the effectiveness and efficiency of the product. Given the massive amount of data being collected, the hope of this assessment was to initiate a communication process to demonstrate the potential value of web GIS and spatial analysis to a wider audience. The question remains; is it relatively unique to display data side-by-side among cancer centers that provides the ability to view data for various datasets and geographical characteristics?

LionVu 2.0 fills a niche in the health community by giving a practitioner the ability to visualize and utilize health data. In the era of big data, there is a need for sharing health data with the public while preserving privacy. LionVu 2.0 presents health-related data spatially in a way that allows people the ability to visualize health data for a given area, while utilizing available health data, at various levels of aggregations and geographical scales (i.e., state, county, zip code, etc.). LionVu 2.0 aims to bridge the gap between data availability and utilization by providing a tool for health practitioners to visualize and utilize health data at the county level, which is small enough to see a difference in a given state, but large enough to minimize data privacy concerns. With the widespread usage of electronic data with various geographical scales there is a need for sharing data that can be used to benefit public health to improve the quality of life. As a result, cancer population researchers are increasingly using geospatial analysis techniques (i.e., exposure assessment, identify spatial associations, proximity analysis, cluster detection and descriptive mapping) to visualize these datasets [27]. LionVu 2.0 includes the most recently available cancer data and includes a wide variety of datasets that can be utilized by anybody who has online access. Although the focus is just on Pennsylvania and the catchment area, it is possible to reuse the code for a wider geographical area. The side-by-side functionality also allows the health practitioner the ability to visualize and compare maps from different layers or datasets. The results of the assessment show that LionVu 2.0 meets the needs by giving the users the ability to provide feedback for further improvements to LionVu 2.1. Based on participants’ feedback, the radio buttons were converted to seven dropdown menus, allowing users to adjust the choropleth map displays. In addition, we uncovered feedback to address how to best respond to the issues uncovered by this assessment and ensure that LionVu 2.1 will meet the needs of its health-care professionals and identified new audiences, such as students.

## Figures and Tables

**Table 1.** Median score of participatory GIS usability scale responses between academics and professionals (10 Responses).

	User Interface	Interaction with the Web Maps	Learnability	Reliability	Communication
**Total (10)**	3	3	4	4	3
**Professionals (4)**	2.5	3	3.5	3.5	3
**Academics (6)**	3	3.5	4	4	3.5

**Table 2.** Performance in median time and success between professionals and academics.

Overall Performance	USCS	Task 1	Task 2	Task 3	Task 4
**Time (s)**	340	343	293	618.5	410.5
**Success**	13%	92%	92%	90%	90%
**Sample**	15	12	12	10	10
**Professionals**					
**Time (s)**	402.5	369	266	532	410.5
**Success**	30%	90%	90%	50%	100%
**Sample**	7	5	5	4	4
**Academics**					
**Time (s)**	325	325	320	1,098	428.5
**Success**	0%	80%	100%	50%	75%
**Sample**	9	7	7	6	6

USCS-United State Cancer Statistics Web Map Task.

**Table 3.** Effectiveness between professionals and academics.

	Professionals			Academics		
Task	Success (%)	Errors	Effectiveness (%)	Success (%)	Errors	Effectiveness (%)
**USCS**	30%	6	5	0%	9	0
**1**	90%	1	90	80%	2	40
**2**	90%	1	90	100%	1	100
**3**	50%	4	12.5	50%	7	7.14
**4**	100%	1	100	75%	4	18.75
**Total**			59.5			33.18

USCS-United State Cancer Statistics Web Map Task.

**Table 4.** Efficiency between professionals and academics.

	Professionals			Academics		
Task	Success (%)	Time (min)	Efficiency (%)	Success (%)	Time (min)	Efficiency (%)
**USCS**	30%	6.70	4.48	0%	5.42	0
**1**	90%	6.15	14.63	80%	5.42	14.76
**2**	90%	4.43	20.31	100%	5.33	18.76
**3**	50%	8.89	5.62	50%	18.3	2.73
**4**	100%	6.84	14.61	75%	7.14	10.48
**Total**			11.93			6.42

USCS-United State Cancer Statistics Web Map Task.

## Appendix A

**Table A1.** LionVu 2.0 Usability Survey Questions.

Section	Question
A. Demographic Questions:	1. Please provide your current age in years. 2. Please provide your racial identify. 3. Please provide your ethnic identity. 4. Please provide your current gender. 5. What is your highest level of education?
B. Employment Characteristics, Prior to the COVID-19 Outbreak:	6. Please specify the locations where you were employed, prior to the COVID-19 outbreak. 7. Please specify employee classifications, prior to the COVID-19 outbreak. 8. Please specify official job title, prior to the COVID-19 outbreak (i.e., Research support technologist). 9. What was your primary organization of employment, prior to the COVID-19 outbreak (i.e., Penn State, Pennsylvania Department of Health, etc.)? 10. Please rate your proficiency (e.g., NIH competencies proficiency scale) with using any web mapping tools.
C. Web Mapping Comparison to United States Cancer Statistics:	11. From the rate of new cancers in the United States web map, what was the age-adjusted rate of new cancers within Pennsylvania, 2012–2016? 12. What are some limitations of the United States web map? 13. What are some strengths of the United States web map? 14. What information do expect to see with the web mapping tools, like the United States web map? 15. What capabilities of the United States web map are important to you?
D. Task 1: Using LionVu 2.0 prototype, determine Population Densities and Hispanic Populations within Luzerne County (hint: use catchment area: Demographics thematic layers, legend, and county popups, except where noted).	16. What is the population density (catchment area: Demographics) of Luzerne County (person/km^2^)? 17. What bin or color value is Luzerne County Hispanics (catchment area: Demographics)? 18. How did you determine the population density and color value? 19. What are your interpretations of population density across Pennsylvania (Pennsylvania: Demographics)? 20. How could the map interface in LionVu 2.0 be improved to help illustrate the demographic characteristics across Pennsylvania (Pennsylvania: Demographics)?
E. Task 2: Using LionVu 2.0 prototype, determine colorectal cancer mortality and late stage percentage within Centre County (hint: use catchment area: Cancer thematic layers, legend, and county popups, except where noted).	21. What is the age-adjusted mortality rate (per 100,000 people) of colorectal cancer (catchment area: Cancer) in Centre County? 22. What is the percentage of late stage colorectal cancer (catchment area: Cancer) in Centre County? 23. How did you determine the colorectal cancer mortality rate and late stage percentage? 24. What are your interpretations of colorectal cancer mortality across Pennsylvania (Pennsylvania: Cancer)? 25. How could the map interface in LionVu 2.0 be improved to help illustrate the cancer characteristics across Pennsylvania (Pennsylvania: Cancer)?
F. Task 3: Using LionVu 2.0 prototype, determine provider characteristics, within Lancaster County (hint: use catchment area: Cancer thematic layers, legend, and county popups, except where noted).	26. What is the density of endoscopy providers (catchment area: Health care facility and area) within Lancaster County (providers/km^2^ per 1000 people)? 27. From the catchment area: Health care facility and area layers, what provider is not found within Lancaster County? 28. How did you determine the selected provider characteristics? 29. How could the map interface in LionVu 2.0 be improved to help illustrate the health care facilities and area characteristics across Pennsylvania (Pennsylvania: Health care facilities and area)? 30. What are your interpretations of the density of endoscopy providers (Pennsylvania: Health care facilities and area—left display) relative to colorectal cancer mortality rates (Pennsylvania: Cancer—right display) across Pennsylvania?
G. Task 4: Using LionVu 2.0 prototype, determine selected characteristics using the side-by-side functionality, within Centre and Luzerne Counties (hint: use both catchment area: Behavioral risks—left display and catchment area: Socioeconomics—right display thematic layers and county popups, except where noted).	31. Using the side-by-side functionality, what is the adult obesity (catchment area: Behavioral risks—left display) and poverty percentages (catchment area: Socioeconomics—right display) in Luzerne County? 32. Using the side-by-side functionality, what is the mammogram screening (catchment area: Behavioral risks—left display) and rurality percentages (Catchment area: Socioeconomics—right display) in Centre County? 33. How did you determine the selected behavioral risks and socioeconomic characteristics? 34. How could the map interface in LionVu 2.0 be improved to help illustrate the behavioral risk characteristics across Pennsylvania (Pennsylvania: Behavioral risks)? 35. How could the map interface in LionVu 2.0 be improved to help illustrate the socioeconomic characteristics across Pennsylvania (Pennsylvania: Socioeconomics)?
H. Rate on your experience using the LionVu 2.0 Prototype (scale: Strongly disagree—strongly agree), based on the participatory GIS usability scale.	36. It is easy to move through different parts of the user interface (Domain: User interface). 37. I can easily access information displayed in the map (Domain: Interaction with the web maps). 38. It is easy to remember how to perform tasks (Domain: Learnability). 39. The system is reliable (Domain: Reliability). 40. The maps are easy to understand (Domain: Communication).
I. Purpose, Data, Documentation, and Functionality Feedback Questions.	41. What do you see as the primary purpose of the LionVu 2.0 prototype? 42. Please specify, what data would you want to remove from the LionVu 2.0 prototype? 43. Please specify, what data would you want to add to the LionVu 2.0 prototype? 44. How can the documentation (read me first), in the LionVu 2.0 prototype, be revised and improved for better comprehension? 45. Please elaborate on your thoughts about what kind of functionality do you consider to be essential for LionVu 2.0.
J. Strengths, Limitations, and General Comments.	46. Other than data, documentation, and functionality, please provide some limitations about the LionVu 2.0 prototype. 47. Other than data, documentation, and functionality, please provide some strengths about the LionVu 2.0 prototype. 48. Please elaborate, whether the LionVu 2.0 Prototype provides you the tools you need to do your job well. 49. Do you have any other feedback, about the LionVu 2.0 Prototype, which was not covered in this usability assessment? 50. Do you have any other comments about this questionnaire or REDCap?
